# Enzymes Catalyzing the TCA- and Urea Cycle Influence the Matrix Composition of Biofilms Formed by Methicillin-Resistant *Staphylococcus aureus* USA300

**DOI:** 10.3390/microorganisms6040113

**Published:** 2018-10-29

**Authors:** Sarah De Backer, Julia Sabirova, Ines De Pauw, Henri De Greve, Jean-Pierre Hernalsteens, Herman Goossens, Surbhi Malhotra-Kumar

**Affiliations:** 1 Department of Medical Microbiology, Vaccine & Infectious Diseases Institute, University of Antwerp, 2610 Wilrijk, Belgium; sarah.debacker@uantwerpen.be (S.D.B.); jus16@dsmz.de (J.S.); ines.depauw@uantwerpen.be (I.D.P.); herman.goossens@uantwerpen.be (H.G.); 2 Structural & Molecular Microbiology, VIB-VUB Center for Structural Biology, 1050 Brussels, Belgium; henri.de.greve@vub.be; 3 Structural Biology Brussels, Vrije Universiteit Brussel, 1050 Brussels, Belgium; 4 Viral Genetics, Vrije Universiteit Brussel, 1050 Brussels, Belgium; jphernal@vub.ac.be

**Keywords:** MRSA, MSSA, *SCCmec*, *argH*, sdhB, sdhA, fumC, tricarboxylic acid cycle, *fnbA*, *fnbB*, *sarA*, arginine, fumarate, malate

## Abstract

In methicillin-sensitive *Staphylococcus aureus* (MSSA), the tricarboxylic acid (TCA) cycle is known to negatively regulate production of the major biofilm-matrix exopolysaccharide, PIA/PNAG. However, methicillin-resistant *S. aureus* (MRSA) produce a primarily proteinaceous biofilm matrix, and contribution of the TCA-cycle therein remains unclear. Utilizing USA300-JE2 Tn-mutants (NARSA) in genes encoding TCA- and urea cycle enzymes for transduction into a prolific biofilm-forming USA300 strain (UAS391-Ery^s^), we studied the contribution of the TCA- and urea cycle and of proteins, eDNA and PIA/PNAG, to the matrix. Genes targeted in the urea cycle encoded argininosuccinate lyase and arginase (*argH*::Tn and *rocF*::Tn), and in the TCA-cycle encoded succinyl-CoA synthetase, succinate dehydrogenase, aconitase, isocitrate dehydrogenase, fumarate hydratase class II, and citrate synthase II (*sucC*::Tn, *sdhA/B*::Tn, *acnA*::Tn, *icd*::Tn, *fumC*::Tn and *gltA*::Tn). Biofilm formation was significantly decreased under no flow and flow conditions by *argH::*Tn, *fumC::*Tn*,* and *sdhA/B::*Tn (range OD_492_ 0.374−0.667; integrated densities 2.065−4.875) compared to UAS391-Ery^S^ (OD_492_ 0.814; integrated density 10.676) (*p* ≤ 0.008). Cellular and matrix stains, enzymatic treatment (Proteinase K, DNase I), and reverse-transcriptase PCR-based gene-expression analysis of fibronectin-binding proteins *(fnbA/B)* and the staphylococcal accessory regulator *(sarA)* on pre-formed UAS391-Ery^s^ and Tn-mutant biofilms showed: (i) < 1% PIA/PNAG in the proteinaceous/eDNA matrix; (ii) increased proteins under no flow and flow in the matrix of Tn mutant biofilms (on average 50 and 51 (±11)%) compared to UAS391-Ery^s^ (on average 22 and 25 (±4)%) (*p* < 0.001); and (iii) down- and up-regulation of *fnbA/B* and *sarA*, respectively, in Tn-mutants compared to UAS391-Ery^S^ (0.62-, 0.57-, and 2.23-fold on average). In conclusion, we show that the biofilm matrix of MRSA-USA300 and the corresponding Tn mutants is PIA/PNAG-independent and are mainly composed of proteins and eDNA. The primary impact of TCA-cycle inactivation was on the protein component of the biofilm matrix of MRSA-USA300.

## 1. Introduction

Nosocomial and community-acquired infections caused by *Staphylococcus aureus* range from superficial to life-threatening [1]. The pathogenic ability of *S. aureus* is greatly facilitated by its capacity to form biofilms, sessile microbial communities that remain embedded in an extracellular polymeric glycocalyx (matrix) or slime layer [2]. Interestingly, recent studies have highlighted differences in biofilm formation between methicillin-sensitive *S. aureus* (MSSA) and their (multi-) drug resistant counterpart, methicillin-resistant *S. aureus* (MRSA) [3,4]. In MSSA, the primary polysaccharide that forms the biofilm matrix is encoded by the *icaADBC* operon and is known as polysaccharide intercellular adhesin PIA or poly-*N*-acetylglucosamine PNAG [5]. On the other hand, MRSA exhibits a primarily proteinaceous biofilm matrix, with very little contribution of PIA/PNAG [6], that is mediated by adhesins such as the fibronectin binding proteins FnbpA/B [7]. In addition, recent reports also show an important contribution of extracellular DNA (eDNA) to the MRSA biofilm matrix [8]. eDNA in *S. aureus* is released by cell lysis, which has been shown to be dependent on autolysins such as the major autolysin *atl* [9,10], and on the holin/antiholin system *cidA/lrgA* [10,11].

The tricarboxylic acid (TCA) cycle is a central metabolic pathway that generates energy (ATP) and precursors for biosynthesis of macromolecules like 2-oxoglutarate [12]. Its role in regulating PIA/PNAG production in staphylococcal species has been well-studied. In *S. epidermidis*, environmental changes that inhibited TCA-cycle activity also resulted in a massive derepression of PIA biosynthetic genes and increased PIA production [13,14]. This inverse correlation was also confirmed for MSSA in a rabbit catheter model of biofilm infection [15]. However, the contribution of the TCA-cycle, if any, to biofilm formation by MRSA remains unclear, given the primarily protein-based matrix and the lack of studies on TCA-cycle inhibition using fluorocitrate or transposon (Tn) mutants.

In this study, utilizing Tn mutants, biofilm models, and various stains and enzymes, we studied the importance of the TCA- and urea cycle for biofilm formation by MRSA-USA300 and the net contribution of proteins, eDNA and PIA/PNAG, to the matrix.

## 2. Materials and Methods

### 2.1. Bacterial Strains and Growth Conditions

**Figure 1:**
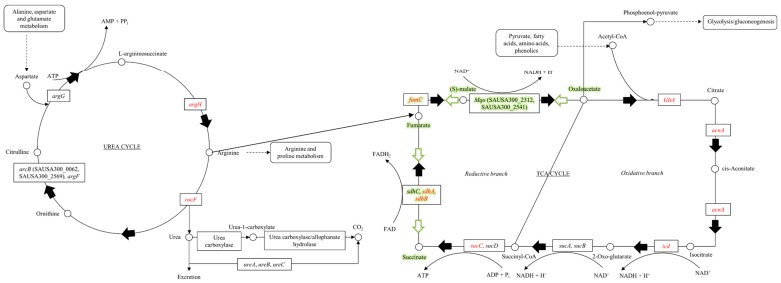
The urea and tricarboxylic acid (TCA)-cycles. Arginine is synthesized via the urea cycle. Carbamoyl phosphate reacts with ornithine to generate citrulline. Addition of aspartate to citrulline creates L-argininosuccinate. ATP is cleaved to AMP and pyrophosphate to drive this reaction forward. Arginine is cleaved off of L-argininosuccinate by the enzyme encoded by *argH* and can be used for protein synthesis. Hydrolysis of arginine generates ornithine and urea. Fumarate is the other product of the ArgH-catalyzed reaction and can be used in the TCA-cycle. Acetyl-CoA derived from pyruvate and other catabolic pathways enters the TCA-cycle. The acetyl group condenses with four-carbon oxaloacetate to produce citrate. Citrate rearranges to isocitrate, which is decarboxylated and forms NADH + H^+^ by transferring 2H^+^ + 2e^−^. 2-Oxoglutarate is decarboxylated and transfers 2H^+^ + 2e^−^ to form NADH + H^+^, while incorporating CoA to form succinyl-CoA. Succinate forms fumarate by transferring 2H^+^ + 2e^−^ resulting in FADH_2_. Water is incorporated, and oxaloacetate is formed when 2H^+^ + 2e^−^ are transferred to form NADH + H^+^. The pathway marked in green highlights the proposed model for NADH reoxidation (Arnon-Buchanan cycle).

The strains used in this study are shown in Table 1. *Bursa aurealis* transposon (Tn) insertion mutations encoding functionally non-redundant TCA- and urea cycle enzymes (Figure 1) in USA300-JE2 were obtained from the Nebraska Transposon Mutant Library (NTML, www.beiresources.org) [16]. Parental strains UAS391, UAS391-Ery^S^ (erythromycin resistance cured UAS391), and JE2 are all MRSA belonging to the highly virulent and widespread clonal lineage, USA300. These, as well as Tn insertion mutants, were routinely grown on Brain-Heart infusion (BHI; Becton, Dickinson and Company, Franklin Lakes, NJ, USA) supplemented with 0.1% D(+)-glucose monohydrate (Merck Millipore, Billerica, MA, USA) and BHI Bacto™ agar (Becton, Dickinson and Company, USA) for biofilm, transduction and complementation experiments. Lysogeny broth (LB; Becton, Dickinson and Company, USA) was used for *Escherichia coli*. For the Tn-carrying *S. aureus* transductants with the erythromycin resistance marker *ermB*, 5 or 10 µg/mL erythromycin (Sigma-Aldrich^®^, Merck KGaA, St. Louis, MO, USA) was supplemented to the growth medium. 

### 2.2. Transduction Experiments

Nine *bursa aurealis* Tn mutations in candidate genes of the TCA- and urea cycles were transduced with phage Φ11 from USA300-JE2 to UAS391-Ery^S^. Gene knockouts in UAS391-Ery^S^ (*argH*::Tn, *icd*::Tn, *acnA*::Tn, *gltA*::Tn, *rocF*::Tn, *fumC*::Tn, *sdhA*::Tn, *sdhB*::Tn, and *sucC*::Tn) were constructed through phage Φ11-mediated homologous recombination of the chromosomal UAS391-Ery^S^ genes with mutated alleles carried by NTML USA300-JE2 Tn insertion mutants, as described in Table 1 [17].

### 2.3. Growth Rate Analysis

To exclude the possibility of changes in biofilm mass due to a pleiotropic effect on the bacterial growth rate, an overnight grown culture of the Tn mutants or UAS391-Ery^S^ was diluted until a concentration of 0.5 McFarland and 20 µL was added to 180 µL fresh BHI-medium in a 96-well microtiter plate (CELLSTAR^®^96 Well Plate Flat Bottom (polystyrene), Greiner Bio-One, Austria). The optical density of each well was measured with a spectrophotometer (MultiSkan™ GO Microplate Spectrophotometer, Thermo Fisher Scientific Inc., Waltham, MA, USA) using SkanIt™ software during a course of 24 h at 37 °C (measurements were taken every 15 min, at 600 nm with shaking at 5 Hz and an amplitude of 15 mm). Growth rates were calculated based on the exponential portion of the curve, the maximum culture density, and the duration of the growth lag phase using GrowthRates software [23]. In total, 96 measurements were made and the growth pattern of each mutant or UAS391-Ery^S^ was measured in 8 different wells.

### 2.4. Quantitative Biofilm Assay under Static (No Flow) Conditions

UAS391-Ery^S^, Tn and complemented mutants were studied as 24 h-old biofilms under flow or no flow conditions as described [18] with one modification; washing to remove planktonic bacteria was performed by gently submerging the plate in a tub of 1× PBS (Thermo Fisher Scientific Inc., Waltham, MA, USA). OD values were measured at 492 nm (Multiskan FC photometer, Thermo Fisher Scientific Inc., Waltham, MA, USA), normalized to the blank and compared to simultaneously run MRSA ATCC reference strains 6538 and 5374 (positive and negative control, respectively), as well as UAS391-Ery^S^. The assay was performed on three distinct days, each time on three different plates and control strains, UAS391-Ery^S^ as well as Tn and complementation mutants on the same plate were added in 6 different wells.

### 2.5. Quantitative Biofilm Assay under Flow (Dynamic) Conditions

All Tn and complemented mutants as well as UAS391-Ery^S^ were also tested for biofilm formation under dynamic conditions in the Bioflux™ system using glass 48-well plates (Fluxion Biosciences Inc., Alameda, CA, USA), as described by Reference [17]. Imaging was performed with a high-end fluorescence Carl Zeiss™ microscope (Axio Observer^®^ with Cell Observer SD, ApoTome.2, LSM710, Göttingen, Germany) using ZEN pro 2012 software (Zeiss Efficient Navigation^®^, Göttingen, Germany). Actual fluorescence quantification of the obtained images was performed using the program ImageJ (Image Processing and Analysis in Java), which measured integrated density (http://imagej.nih.gov/). The assay was performed on two distinct days. Control strains, UAS391-Ery^S^, as well as Tn and complemented mutants on the same plate were added in duplicate.

### 2.6. Analysis of Biofilm Matrix Composition

Additionally, UAS391-Ery^S^, Tn and complemented mutants grown under flow and no flow conditions were studied for differences in cell densities and viability, as well as matrix composition using LIVE/DEAD™ (BacLight™ Bacterial Viability Kit), SYPRO^®^ Ruby (FilmTracer™ SYPRO^®^ Ruby Biofilm Matrix Stain), or wheat germ agglutinin (WGA) (Wheat Germ Agglutinin, Texas Red™-X Conjugate) fluorescent stains (Thermo Fisher Scientific Inc., Waltham, MA, USA). LIVE/DEAD™ stain consists of SYTO™ 9 which stains the entire cell mass green followed by propidium iodide which will only stain the dead or dying cells with a compromised membrane (red). WGA Texas Red™-X Conjugate binds to sialic acid and *N*-acetylglucosaminyl residues of PIA/PNAG, and FilmTracer™ SYPRO^®^ Ruby Biofilm Matrix Stain labels most classes of proteins, such as glycoproteins, phosphoproteins, lipoproteins, calcium binding proteins, and fibrillar proteins. Briefly, after 24 (no flow) or 17 h (flow) growth and rinsing with either 1× PBS (no flow) or 0.9% sodium chloride (flow) to remove planktonic cells, biofilms were stained and microscopically visualized using the ImageJ program for data measurements, as explained before. Under no flow conditions (used for quantification), the assay was performed on three distinct days, each time on three different plates and control strains, UAS391-Ery^S^ as well as mutants on the same plate were added in 6 different wells. Under flow conditions (used for visualization), the assay was performed on two distinct days, with control strains, UAS391-Ery^S^ as well as Tn and complemented mutants added *in duplo* on the same plate. Percentages compared to UAS391-Ery^S^ were calculated as µm² area covered. In order to quantify the proportion of protein and eDNA in the biofilm matrix, pre-formed biofilms, grown under flow or no flow conditions, were rinsed once with either 1X PBS (no flow) or 0.9% sodium chloride (flow) incubated for 2 (no flow) or 5h (flow) at 37 °C with Proteinase K (Sigma-Aldrich^®^, Merck KGaA, St. Louis, MO, USA) (100 µg/mL in culture medium with 10 mM Tris-HCl, pH 7.5) or DNaseI (100 U/mL in culture medium) (Sigma-Aldrich^®^, Merck KGaA, St. Louis, MO, USA). Control wells were treated with the appropriate buffer. Afterwards, the wells were washed and stained, as described before. Under no flow conditions (used for quantification), the assay was performed on three distinct days, each time on three different plates and control strains, UAS391-Ery^S^ as well as mutants on the same plate were added in 6 different wells. Under flow conditions (used for visualization), the assay was performed on two distinct days, with control strains, UAS391-Ery^S^ as well as Tn, and complemented mutants added in duplo on the same plate.

### 2.7. Relative Gene Expression Analysis

To measure the impact of the Tn insertion in the target gene on the expression of the global regulator *sarA* and on the fibronectin-binding proteins encoded by *fnbA/B*, 24h-old no flow biofilms of Tn mutants and of UAS391-Ery^S^ were mechanically disrupted using bead beating *(*FastPrep^®^-24 classic homogenization instrument, MP Biomedicals, Irvine, CA, USA). Total RNA was isolated (Masterpure™ Complete DNA and RNA Purification kit, Epicentre^®^, Madison, WI, USA), 1 μg RNA was purified (Turbo DNA-free™, Ambion^®^, Thermo Fisher Scientific Inc., Waltham, MA, USA) and first-strand cDNA was synthesized using random primers (Reverse Transcription System, Promega Corporation, Madison, WI, USA). Reverse transcriptase-PCR (RT-PCR) was performed (StepOnePlus™ system, Applied Biosystems^®^, Thermo Fisher Scientific Inc., Waltham, MA, USA) with *Power* SYBR™ Green PCR Master Mix (Thermo Fisher Scientific Inc., Waltham, MA, USA). Gene-specific primers are listed in Supplementary Table S1. For data normalization, housekeeping gene *gyrB* (SAUSA300_0005) was used as an internal reference and the fold change in gene expression was calculated using the comparative C_t_ method (2^−ΔΔCt^).

### 2.8. Complementation Experiments

To confirm that the changed biofilm phenotype of the Tn mutants was caused by inactivation of the target gene and not because of secondary mutations in the genome, complementation by a cloned wild type copy of the target genes was performed. Briefly, total genomic DNA of UAS391-Ery^S^ was purified and the *argH*, *acnA*, *icd*, *gltA*, *fumC*, *sucC*, *sdhA*, *sdhB,* and *rocF* genes with a 25–26 bp. overlap corresponding to the nucleotide sequences flanking the EcoRI site of the shuttle vector pALC2073, were amplified, as described in Reference [17]. For genes in an operon, the distal genes were also included in the PCR fragments that were used for complementation. Primers are listed in Supplementary Table S1. PCR-fragments were cloned (2× Gibson Assembly^®^ Master Mix, New England BioLabs^®^ Inc., Ipswich, MA, USA) in the EcoRI-linearized (New England BioLabs^®^ Inc., Ipswich, MA, USA) pALC2073 vector, transformed into recombination-impaired CaCl_2_-competent *E. coli* DH5α and transformants were selected on LB supplemented with carbenicillin (100 µg/mL). Transformants were checked by Sanger sequencing using pALC2073 vector primers TetR2, pALC-2, and internal gene sequence primers of interest (Supplementary Table S1). Constructs were first introduced by electroporation into the restriction-deficient *S. aureus* host RN4220 to adapt plasmid DNA from *E. coli* to *S. aureus* modifications. Transformants were selected on LB agar plates supplemented with 10 μg/mL chloramphenicol (Sigma-Aldrich^®^, Merck KGaA, St. Louis, MO, USA). Subsequently, plasmid DNAs isolated from this strain were introduced by electroporation into the corresponding UAS391-Ery^S^ Tn mutants and the transformants were again selected on LB medium supplemented with 10 μg/mL chloramphenicol. The expression of the cloned genes was induced by adding 0.1 μg/mL anhydrotetracycline (Sigma-Aldrich^®^, Merck KGaA, St. Louis, MO, USA) to the growth media.

### 2.9. Statistical Analysis

Biomass quantification in the dynamic flow and no flow assays, as well as growth rate analysis was performed using the R Project software (version 3.1.2.) (R foundation, Vienna, Austria). A Welch two-sample *t*-test or a Wilcoxon Rank Sum test was used when data was either distributed normally or not, based on a Shapiro-Wilk Normality test. *p*-values < 0.05 were considered significant.

## 3. Results

### 3.1. Urea Cycle Mutant argH::Tn, but Not rocF::Tn, Demonstrates a Significantly Decreased Capacity for Biofilm Formation

**Figure 2:**
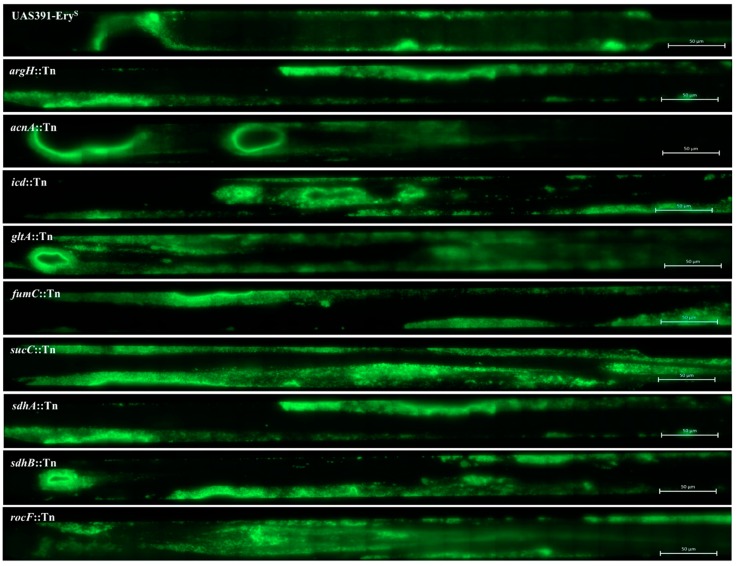
Effect of argininosuccinate lyase (*argH*), aconitate hydratase A (*acnA*), isocitrate dehydrogenase (*icd*), citrate synthase II (*gltA*), fumarate hydratase class II (*fumC*), succinate—CoA ligase (subunit beta) (*sucC*), succinate dehydrogenase (flavoprotein subunit) (*sdhA*), succinate dehydrogenase iron-sulfur protein (*sdhB*), and arginase (*rocF*) Tn mutants on the biofilm phenotype by UAS391-Ery^S^. Flow biofilm images were captured after 17 h growth employing ZEN 2012 software (Zeiss) as 84 combined tile images consisting of one µm² horizontal tiles covering the entire microchannel.

Comparison of average growth rates of *argH*::Tn and *rocF*::Tn mutants with UAS391-Ery^S^ showed no decrease in growth rates (*p* = 0.158 and *p* = 0.207, respectively) (Table 2 and Supplementary Figure S1). Next, no flow and flow biofilms formed by the *argH*::Tn and *rocF*::Tn mutants were quantified compared to UAS391-Ery^S^. The *argH*::Tn mutant showed a significant decrease in biofilm formation both in the no flow (*p* < 0.001) and in the flow model (*p* = 0.008), compared to UAS391-Ery^S^ (Table 2 and Figure 2). However, a similarly significant decrease was not observed with the *rocF::Tn* mutant (*p* ≥ 0.103) (Table 2 and Figure 2). The biomass under flow and no flow conditions increased for *argH::*Tn upon complementation with pGV5990 to quantities comparable to UAS391-Ery^S^ (*p* ≤ 0.045), while the complemented *rocF::*Tn (with pGV6003) showed no change in biofilm formation, as compared to *rocF*::Tn mutant or to UAS391-Ery^S^ (*p* ≥ 0.057) (Table 2).

### 3.2. TCA-Cycle Mutants, fumC::Tn, sdhA::Tn and sdhB::Tn, but not acnA::Tn, icd::Tn, gltA::Tn and sucC::Tn, Demonstrate a Significantly Decreased Capacity for Biofilm Formation

None of the 7 mutants tested (*acnA*::Tn, *icd*::Tn, *gltA*::Tn, *fumC*::Tn, *sucC*::Tn, *sdhA*::Tn, and *sdhB*::Tn) displayed impaired growth rates (*p* = 0.554) (Table 2 and Supplementary Figure S1). Biofilm formation under no flow conditions decreased by 1.5- to 2.2-fold in *fumC*::Tn (*p* < 0.001) and *sdhA*::Tn (*p* < 0.001), as compared to UAS391-Ery^S^. Biofilm decrease was only 1.2-fold in *sdhB*::Tn (*p* = 0.048). However, no significant decrease in biofilm formation was detected in the *acnA*::Tn, *gltA*::Tn, *sucC*::Tn, and *icd*::Tn mutants (*p* ≥ 0.059) (Table 2). Under flow conditions, the *fumC*::Tn, *sdhA*::Tn and *sdhB*::Tn mutants also showed the largest decrease in biomass, compared to UAS391-Ery^S^ (*p* ≤ 0.012). *AcnA*::Tn formed less biofilm under flow conditions (*p* = 0.041), despite not displaying any effect under no flow conditions while *gltA*::Tn, *sucC*::Tn, and *icd*::Tn mutants showed no significant changes in biofilm formation (*p* ≥ 0.101) (Table 2 and Figure 2). Biofilm formation by Tn mutants complemented with the corresponding intact gene (Table 1), showed no differences in biofilm formation, as compared to UAS391-Ery^S^ under no flow (*p* = 0.423) or flow conditions (*p* = 0.053) (Table 2).

### 3.3. Fluorescent Staining of the Biofilm Matrix Reveals That the Protein Component Is Decreased in TCA- and Urea Cyle Mutants

**Figure 3:**
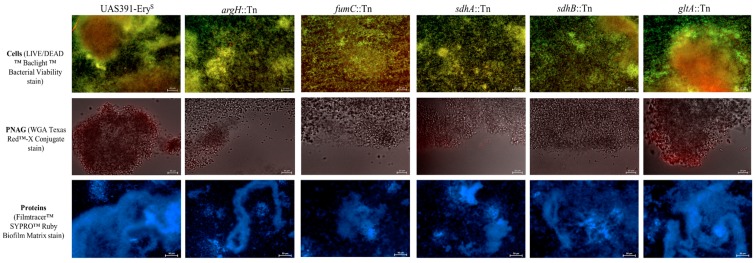
Fluorescence microscopy observations of no flow biofilm matrix structure obtained from UAS391-Ery^S^, argininosuccinate lyase (*argH*), fumarate hydratase class II (*fumC*), succinate dehydrogenase (flavoprotein subunit) (*sdhA*), succinate dehydrogenase iron-sulfur protein (*sdhB*), and citrate synthase II (*gltA*) Tn mutants. Since microscopy images of *gltA*::Tn, *acnA*::Tn, *icd*::Tn, *sucC*::Tn, and *rocF*::Tn, as well as corresponding complemented mutant biofilms were comparable to each other, the matrix formed by *gltA*::Tn serves as an example picture for all. The top row shows total cells stained with SYTO™ 9 and PI. The middle row shows PNAG stained with WGA Texas Red™-X Conjugate, and is combined with Bright-field imaging. The bottom row shows the protein component stained with FilmTracer™ SYPRO™ Ruby Biofilm Matrix.

Under flow conditions, UAS391-Ery^S^ biofilms showed an average live:dead cell ratio of 61%:39% ± 8%, and a count of 606 ± 15 cells on the total imaged surface area. All 9 Tn mutants (*argH*::Tn, *acnA*::Tn, *icd*::Tn, *gltA*::Tn, *fumC*::Tn, *sucC*::Tn, *sdhA*::Tn, *sdhB*::Tn, and *rocF*::Tn, respectively) showed similar total cell numbers (609 ± 29, respectively) (*p* ≥ 0.881). In contrast, the average live:dead cell ratio changed significantly for *argH*::Tn, *fumC*::Tn, *sucC*::Tn, *sdhA/*B::Tn, and *rocF*::Tn (46%:54% ± 3%) (*p* ≤ 0.001), but not for *acnA*::Tn, *icd*::Tn, and *gltA*::Tn (59%:41% ± 2%) (*p* ≥ 0.168) (Table 2 and Figure 3). Complemented strains showed no significant difference compared to the UAS391-Ery^S^ live:dead cell ratio (on average 59%:41% ± 9%) (p ≥ 0.928). Staining with WGA Texas Red™-X conjugate showed < 1% total area coverage by PIA/PNAG for UAS391-Ery^S^, as well as for all 9 Tn mutants and for their corresponding complemented strains (*p* ≥ 0.943) (Table 2 and Figure 3). In contrast, WGA staining of the PIA/PNAG producing MSSA control strain ATCC^®^ 25923™ (image not shown) gave a total area coverage of 91% ± 2% (Table 2). Staining with FilmTracer™ SYPRO^®^ Ruby Biofilm Matrix Stain showed an average area coverage decrease of 48% ± 19% compared to that of UAS391-Ery^S^ for *argH*::Tn, *acnA*::Tn, *icd*::Tn, *gltA*::Tn, *fumC*::Tn, *sucC*::Tn, *sdhA/B*::Tn, and *rocF*::Tn (*p* ≤ 0.001) (Table 2). Complemented mutants showed similar average protein proportions of the biofilm matrix, as compared to UAS391-Ery^S^ (105% ± 7%) (*p* ≥ 0.194)

### 3.4. Enzymatic Digest Correlates BIOFILM-Defective Transposon Mutants with a Protein- and eDNA-Based Matrix 

**Figure 4:**
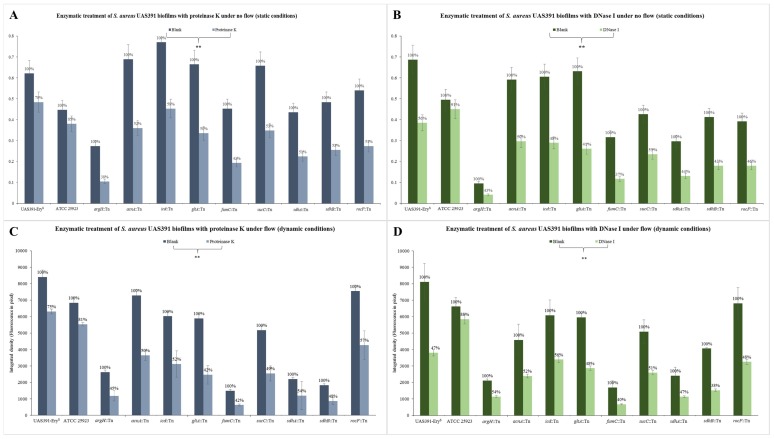
Enzymatic treatment of preformed 24 (no flow) or 17 (flow) h-old biofilms of UAS391-Ery^S^, ATCC^®^ 25923™ and urea or TCA-cycle Tn mutants with 100 µg/mL proteinase K (**A**,**C**) or 100 U/mL DNase I (**B**,**D**) under no flow (no flow) (**A**,**B**) and dynamic (flow) (**C**,**D**) conditions. Blanks refer to incubation in either culture medium with 10 mM Tris-HCl for proteinase K treatment or culture medium for DNase I treatment. Error bars represent 95% confidence intervals. ** refers to *p* < 0.001.

Under no flow conditions, the 24h biomass of UAS391-Ery^S^ grown decreased by 22% ± 3% following proteinase K and 44% ± 5% following DNase I treatment (both *p* < 0.001), while the biomass of ATCC^®^ 25923™ *S. aureus* decreased by only 11% ± 3% and 6% ± 2%, respectively (*p* ≥ 0.134) (Figure 4A,B). Under flow conditions, the 17h biomass of UAS391-Ery^S^ decreased by 25% ± 7% following proteinase K and 53% ± 9% following DNase I treatment (*p* ≤ 0.028), while that of ATCC^®^ 25923™ *S. aureus* decreased by only 19% ± 8% and 12% ± 7%, respectively (*p* ≥ 0.145) (Figure 4C,D). After proteinase K treatment under no flow conditions, *argH*::Tn, *acnA*::Tn, *icd*::Tn, *gltA*::Tn, *fumC*::Tn, *sucC*::Tn, *sdhA/B*::Tn, and *rocF*::Tn showed on average 50% ± 12% reduction in biomass compared to the untreated biofilms of the corresponding mutants (*p* ≤ 0.001) (Figure 4A). Similarly, after proteinase K treatment under flow conditions, there was an average of 51% ± 8% (*p* ≤ 0.05) (Figure 4C). After DNase I treatment under no flow conditions, *argH*::Tn, *acnA*::Tn, *icd*::Tn, *gltA*::Tn, *fumC*::Tn, *sucC*::Tn, *sdhA/B*::Tn, and *rocF*::Tn showed an average 55% ± 10% reduction in biofilm mass as compared to the untreated biofilms of the corresponding mutants. (*p* ≤ 0.005) (Figure 4B). Similarly, after DNase I treatment under flow conditions, there was a reduction of 52% ± 10% respectively (*p* ≤ 0.05) (Figure 4D). Complemented strains showed an average biomass decrease of 23% ± 6% and 48% ± 10% after both treatments under flow and no flow conditions (*p* < 0.001), which is comparable to results obtained with UAS391-Ery^S^.

In all TCA- and urea cycle Tn mutants, *sarA* showed a distinct upregulation (1.35 to 3.63-fold), with the exception of *sucC*::Tn, which showed a 0.92-fold downregulation (Supplementary Figure S2). Overall, a knockout mutation in either the TCA- or urea cycle was associated with a decrease in *fnbA* and *fnbB* expression (0.53- to 0.78-fold and 0.51 to 0.77-fold, respectively) (Supplementary Figure S2).

## 4. Discussion

The biofilm matrix of MRSA-USA300 and corresponding Tn mutants is PIA/PNAG-independent and mainly composed of proteins and eDNA.

Using enzymatic digest and biofilm-matrix staining experiments to assess the contribution of PIA/PNAG, protein, and eDNA to the biofilm matrix of MRSA-USA300, we firstly showed that the biofilm matrix of USA300 UAS391-Ery^S^ and Tn mutants was primarily composed of proteinaceous material and eDNA with < 1% contribution of PIA/PNAG. Interestingly, and in contrast to MSSA, inactivation of the TCA-cycle in MRSA Tn mutants did not result in any increase of PIA/PNAG in the biofilm matrix. In MSSA, decreased TCA-cycle activity was reported to shunt metabolites toward PIA/PNAG production [14]. We have previously whole genome sequenced the UAS391 strain and found an intact functional *icaADBC* operon [19]. These data fully support the results of Pozzi et al. that showed that high level expression of PBP2a—the product of the methicillin resistance gene, *mecA*, harboured on the SCC*mec* element that differentiates MRSA and MSSA—blocks *icaADBC*-dependent polysaccharide biofilm development and promotes formation of proteinaceous biofilms [5]. Of note, *S. aureus* also produces a capsular polysaccharide (type 5 and 8), which has been implicated in biofilm formation [24]. The role of the TCA-cycle in capsular polysaccharide production was demonstrated by Sadykov et al. who showed that in the absence of glucose, the capsular sugar precursor fructose 6-phoshate is synthesized by gluconeogenesis from the TCA-cycle intermediate oxaloacetate [25]. However, the USA300 clonal lineage, including the UAS391 strain, harbors conserved mutations in the *cap5* locus, and does not produce a capsular polysaccharide [26], which also made it easier to exclude the contribution of PIA/PNAG to the biofilm matrix of UAS391 and its Tn mutants.

### 4.1. TCA-Cycle Inactivation Impacts the Protein Component of the Biofilm Matrix of MRSA-USA300

Upon comparison of the net protein contribution to the matrix under no flow and flow conditions, it was clear that the net contribution of proteins to the entire Tn mutant biomass (on average 50% and 51%, respectively) was significantly higher than for UAS391-Ery^S^ (on average 22% and 25%, respectively).

Several studies have reported a role for proteins and eDNA in the *ica*-independent MRSA biofilm phenotype [8,9,27]. Houston et al. demonstrated an important role for eDNA during the primary attachment and early stages of MRSA biofilm formation by employing a Δ*atl* knockout mutant in MRSA isolate BH1CC [9]. These authors also reported that DNaseI impaired biofilm development by MRSA isolates from clonal complex 5 (CC5), CC22 and sequence type 239 (ST239). Moreover, treatment of USA300 biofilms after 6 h and 22 h of growth demonstrated both a significant impact of DNaseI and proteinase K, with the latter having the largest impact on the total biofilm mass [27]. Our results also indicate the possibility of the USA300 biofilm matrix containing other components, as protein and eDNA only accounted for on average 23.5% and 48.5% of the biomass. These might have been teichoic acids associated with the cell wall (cell wall teichoic acid, WTA) or cell membrane (lipoteichoic acid, LTA) [28].

A prior study on an *ica*-knockout MSSA strain RN6390 has shown that, in the presence of citrate, the fibronectin-binding proteins, FnBPA and FnBPB, stimulate biofilm formation by promoting both cell-to-surface and cell-to-cell interactions, which is part of a larger network of virulence factors that are controlled by the staphylococcal accessory regulator, SarA [29]. All urea and TCA-cycle Tn mutants in our study showed a significant downregulation in *fnbA* and *fnbB* gene expression compared to UAS391-Ery^S^, whereas *sarA* was upregulated for all Tn mutants except *sucC*::Tn. SarA has been reported to work synergistically with the two-component *saeRS* system to repress extracellular proteases that would otherwise reduce the accumulation of critical proteins that contribute to the biofilm matrix [30]. Downregulation of *fnbA/*B would potentially lead to a decreased protein biofilm matrix, but upregulation of sarA might neutralize and counteract this effect in the Tn mutants.

### 4.2. Inactivation of Specific TCA-Cycle Genes Is Associated with a High Metabolic Fitness Cost

Using live-dead staining on flow biofilms, we found a significantly higher amount of dead cells in the biofilms formed by *argH*::Tn, *fumC*::Tn, *sdhA*::Tn, *sdhB*::Tn, *sucC*::Tn, and *rocF*::Tn mutants. However, these mutants neither demonstrated attenuated or slower growth on growth curve assays nor were the total number of cells in their respective biofilms significantly different from those in UAS-391-Ery^s^ biofilms. However, Halsey et al. have shown that Tn mutants with mutations in TCA-cycle genes past the 2-oxoglutarate node (*fumC*, *sdhA*, and *sucC*) did not grow at all in their planktonic S. aureus growth assay [31]. The fact that these defects were not detected in our corresponding TCA-cycle Tn mutants might be due to differences in growth media.

A higher number of dead cells found in the biofilms of Tn mutants might be indicative of a high metabolic cost for the bacterium. It is important to note that propidium iodide does not only stain cells with a compromised membrane, but also eDNA. However, the proportion of eDNA in the biofilm matrix, observed by DNase I digestion, did not differ significantly between the Tn mutants (on average 52% and 55% under flow and no flow conditions, respectively) and UAS391-Ery^S^ (on average 44% and 53% under flow and no flow conditions, respectively). Thus, based on our data, inactivation of the TCA-cycle is likely not associated with any change in eDNA biofilm matrix composition.

In conclusion, we identified an important role of the TCA-cycle in mediating biofilm formation, specifically by influencing the matrix composition, in MRSA USA300 biofilms. These metabolic pathway hits require further screening of MRSA of different clonal lineages to confirm commonality of the target mechanisms and eventually yield interesting therapeutic targets.

## Figures and Tables

**Table 1:** Bacterial strains and plasmids used during this study.

Name	Description	Source
*Strains*		
UAS391	Prolific biofilm forming MRSA USA300 strain isolated from a patient with an abscess in a Belgian hospital.	[18,19]
UAS391-Ery^S^	Erythromycin-sensitive variant (loss of *ermC* gene) of *S. aureus* UAS391 obtained by plasmid curing through growth at 44 °C.	[17]
JE2	Plasmid-cured derivative of MRSA USA300 LAC, isolated from a skin and soft tissue infection in a detainee from the Los Angeles County jail.	[16]
RN0450 (NRS135)	MSSA strain derived by successive cycles of UV treatment of *S. aureus* strain NCTC8325, curing it of phages Φ11, Φ12 and Φ13.	NARSA repository
RN0451 (NRS136)	MSSA strain derived from *S. aureus* strain RN0450, lysogenic for phage Φ11.	NARSA repository
RN4220	Generated through UV and chemical mutagenesis of *S. aureus* strain RN0450 and selected for transformability with DNA from *E. coli* (restriction deficient through mutation in *sau1 hsdR*).	NARSA repository
DH5α	*Escherichia coli* cloning strain with multiple mutations (*fhuA2 lacΔU169 phoA glnV44 Φ80’ lacZΔM15 gyrA96 recA1 relA1 endA1 thi-1 hsdR17*) that enable efficient transformation.	Thermo Fisher Scientific Inc., Waltham, MA, USA
ATCC^®^ 6538™	Positive quality control for biofilm formation of *S. aureus* under no flow conditions.	[20]
5374	Negative quality control for biofilm formation of *S. aureus* under no flow conditions.	[18]
ATCC^®^ 25923™	PIA/PNAG-dependent biofilm producing strain used as positive quality control during fluorescent staining and enzymatic treatment of biofilm matrix.	[21]
NE106 (NR-46649)	JE2 Tn mutant (insertion position: 943092) in argininosuccinate lyase (*argH*, SAUSA300_0863; 942072-943451).	NARSA repository
NE134 (NR-46677)	JE2 Tn mutant (insertion position: 2290057) in arginase (*rocF*, SAUSA300_2114; 2289284-2290192).	NARSA repository
NE427 (NR-46970)	JE2 Tn mutant (insertion position: 1985575) in fumarate hydratase, class II (*fumC*, SAUSA300_1801; 1984212-1985597).	NARSA repository
NE491 (NR-47034)	JE2 Tn mutant (insertion position: 1799416) in isocitrate dehydrogenase, NADP-dependent (*icd*, SAUSA300_1640; 1798291-1799559).	NARSA repository
NE569	JE2 Tn mutant (insertion position: 1247122) in succinyl-CoA synthetase, beta subunit (*sucC*, SAUSA300_1138; 1246832-1247998).	NARSA repository
NE594 (NR-47137)	JE2 Tn mutant (insertion position: 1800430) in citrate synthase II (*gltA*, SAUSA300_1641; 1799608-1800729).	NARSA repository
NE626 (NR-47169)	JE2 Tn mutant (insertion position: 1145819) in succinate dehydrogenase, flavoprotein subunit (*sdhA*, SAUSA300_1047; 1145459-1147225).	NARSA repository
NE808 (NR-47351)	JE2 Tn mutant (insertion position: 1147490) in succinate dehydrogenase iron-sulfur subunit (*sdhB*, SAUSA300_1048; 1147225-1148040).	NARSA repository
NE861 (NR-47404)	JE2 Tn mutant (insertion position: 1367722) in aconitate hydratase (*acnA*, SAUSA300_1246; 1367131-1369836).	NARSA repository
*Plasmids*		
pALC2073	Contains the pSK236 vector, with the *tetR*-gene and the *xyl*/*tetO* promotor, originating from pWH35.	[22]
pGV5990	*argH* gene amplified with primers ArgH-1 and ArgH-2 and cloned in the *EcoRI* site of pALC2073 via Gibson cloning.	This study
pGV5992	*gltA* gene amplified with primers GltA-1 and GltA-2 and cloned in the *EcoRI* site of pALC2073 via Gibson cloning.	
pGV5994	*icd* gene amplified with primers Icd-1 and Icd-2 and cloned in the *EcoRI* site of pALC2073 via Gibson cloning.	
pGV5996	*sdhB* gene amplified with primers SdhB-1 and SdhB-2 and cloned in the *EcoRI* site of pALC2073 via Gibson cloning.	
pGV5998	*sucC* gene amplified with primers SucC-1 and SucC-2 and cloned in the *EcoRI* site of pALC2073 via Gibson cloning.	
pGV5999	*gltA* & *icd* genes (operon of two genes; 1st gene *gltA* & 2nd gene *icd*) amplified with primers GltA-1 and Icd-2 and cloned in the *EcoRI* site of pALC2073 via Gibson cloning.	
pGV6000	*sucC* genes (operon of two genes) amplified with primers SucC-1 and SucC-3 and cloned in the *EcoRI* site of pALC2073 via Gibson cloning.	
pGV6001	*sdhA* gene amplified with primer SdhA-1 and SdhA-2 and cloned in the *EcoRI* site of pALC2073 via Gibson cloning.	
pGV6002	*sdhA* (2nd gene in operon of 3 genes) & *sdhB* (3rd gene in operon of 3 genes) genes amplified with primers SdhA-1 and SdhB-2 and cloned in the *EcoRI* site of pALC2073 via Gibson cloning.	
pGV6003	*rocF* gene amplified with primers RocF-1 and RocF-2 and cloned in the *EcoRI* site of pALC2073 via Gibson cloning.	
pGV6005	*fumC* gene amplified with primers FumC-1 and FumC-2 and cloned in the *EcoRI* site of pALC2073 via Gibson cloning.	
pGV6007	*acnA* gene amplified with primers AcnA-1 and AcnA-2 and cloned in the *EcoRI* site of pALC2073 via Gibson cloning.	

**Table 2:** Overview of results. Quantification of formed biofilm mass (optical density, no flow assay; integrated density, dynamic assay), growth rate (growth curve assay), ratio live:dead cells (Syto™ 9 Green Fluorescent Acid and propidium iodide stain), protein component (Filmtracer™ SYPRO™ Ruby Biofilm Matrix stain), and PIA/PNAG component (WGA Texas Red™-X Conjugate stain). Standard deviations are mentioned next to each value (±) and the percentage value compared to UAS391-Ery^S^ is mentioned between brackets. NT refers to not tested.

Strain	Optical Density (OD_492_)	Integrated Density (Fluorescence in Pixels)	Growth Rate (min^−1^)	Ratio Live:Dead Cells (%)	Protein Component (%)	PIA/PNAG Component (%)
UAS391-Ery^s^	0.814 ± 0.14 (100%)	11,301 ± 61 (100%)	0.157 ± 0.01	61:39 ± 3:1	100 ± 3	100 ± 6
ATCC^®^ 25923™	NT	NT	NT	58:42 ± 13:4	34 ± 8	835 ± 2
*argH*::Tn	0.504 ± 0.07 (62%)	3653 ± 45 (32%)	0.161 ± 0.01	44:56 ± 2:1	48 ± 10	110 ± 15
*argH*::Tn with pGV5990	0.828 ± 0.15 (102%)	9389 ± 66 (83%)	NT	59:41 ± 16:5	83 ± 2	106 ± 16
*acnA*::Tn	0.782 ± 0.11 (96%)	651 ± 036 (58%)	0.163 ± 0.01	61:39 ± 2:1	64 ± 3	77 ± 4
*acnA*::Tn with pGV6007	0.805 ± 0.15 (99%)	773 ± 57 (68%)	NT	60:40 ± 10:3	92 ± 16	101 ± 11
*icd*::Tn	0.838 ± 0.13 (103%)	8264 ± 16 (73%)	0.166 ± 0.01	58:42 ± 13:4	67 ± 4	110 ± 17
*icd*::Tn with pGV5994	1.143 ± 0.12 (141%)	8308 ± 163 (74%)	NT	57:43 ± 15:5	91 ± 3	100 ± 20
*icd*::Tn with pGV5999	1.248 ± 0.17 (153%)	8076 ± 133 (72%)	NT	54:46 ± 19:7	96 ± 22	101 ± 9
*gltA*::Tn	0.789 ± 0.11 (97%)	7911 ± 11 (70%)	0.157 ± 0.01	57:43 ± 18:6	61 ± 10	91 ± 5
*gltA*::Tn with pGV5992	1.102 ± 0.12 (135%)	9616 ± 25 (85%)	NT	61:39 ± 5:2	93 ± 0	100 ± 12
*gltA*::Tn with pGV5999	1.055 ± 0.10 (130%)	8149 ± 27 (72%)	NT	59:41 ± 7:2	89 ± 9	100 ± 2
*fumC*::Tn	0.374 ± 0.07 (46%)	2065 ± 42 (18%)	0.162 ± 0.01	43:57 ± 1:0	49 ± 0	122 ± 7
*fumC*::Tn with pGV6005	0.941 ± 0.14 (116%)	7662 ± 188 (68%)	NT	66:34 ± 10:3	80 ± 5	100 ± 13
*sucC*::Tn	0.847 ± 0.17 (104%)	7506 ± 58 (67%)	0.150 ± 0.01	47:53 ± 19:6	54 ± 3	103 ± 17
*sucC*::Tn with pGV5998	0.804 ± 0.11 (99%)	8715 ± 10 (77%)	NT	65:35 ± 15:5	96 ± 7	101 ± 16
*sucC*::Tn with pGV6000	1.041 ± 0.13 (128%)	7599 ± 159 (67%)	NT	68:32 ± 11:4	83 ± 2	101 ± 5
*sdhA*::Tn	0.537 ± 0.11 (66%)	3835 ± 60 (34%)	0.162 ± 0.01	47:53 ± 5:2	33 ± 3	89 ± 8
*sdhA*::Tn with pGV6001	1.002 ± 0.10 (123%)	9002 ± 11 (80%)	NT	51:49 ± 6:2	78 ± 8	101 ± 6
*sdhA*::Tn with pGV6002	1.071 ± 0.11 (132%)	7252 ± 092 (64%)	NT	53:47 ± 4:1	92 ± 10	100 ± 11
*sdhB*::Tn	0.667 ± 0.10 (82%)	4875 ± 056 (43%)	0.159 ± 0.01	48:52 ± 5:2	44 ± 18	84 ± 7
*sdhB*::Tn with pGV5996	0.908 ± 0.12 (112%)	11,057 ± 129 (98%)	NT	66:34 ± 6:2	106 ± 21	100 ± 13
*sdhB*::Tn with pGV6002	1.140 ± 0.09 (140%)	8455 ± 65 (75%)	NT	49:51 ± 5:2	92 ± 3	100 ± 17
*rocF*::Tn	0.765 ± 0.12 (94%)	7586 ± 15 (67%)	0.163 ± 0.01	49:51 ± 5:2	51 ± 11	89 ± 19
*rocF*::Tn with pGV6003	0.878 ± 0.09 (108%)	8342 ± 21 (74%)	NT	65:35 ± 12:4	84 ± 3	101 ± 8

## Supplementary Materials

The following are available online at http://www.mdpi.com/2076-2607/6/4/113/s1, Figure S1: Background absorption-corrected 24h growth curves for UAS391-Ery^S^ as well as *argH*::Tn, *acnA*::Tn, *icd*::Tn, *gltA*::Tn, *fumC*::Tn, *sucC*::Tn, *sdhA*::Tn, *sdhB*::Tn and *rocF*::Tn mutants. Error bars in corresponding color represent the 95% confidence interval per strain; Figure S2: Relative quantification of *fnbA*, *fnbB* and *sarA* gene expression in UAS391-Ery^S^ and the corresponding TCA- and urea cycle knockout mutants, normalized to *gyrB* expression and calculated using the Comparative C_t_ Method (2^−ΔΔCt^). Gene expression in UAS391-Ery^S^ was taken as baseline 0; Table S1: Primers used during this study. Primers were designed against *S. aureus* strain USA300-UAS391 (#CP007690.1).
