# Supplementary material for: Enzymes Catalyzing the TCA- and Urea Cycle Influence the Matrix Composition of Biofilms Formed by Methicillin-Resistant Staphylococcus aureus USA300
Source: Microorganisms. 2018 Oct 29;6(4):113. doi: 10.3390/microorganisms6040113 (PMC6313315; doi:10.3390/microorganisms6040113)
Supplement: Supplementary file 1 [file microorganisms-06-00113-s001.pdf]

Supplementary Materials:

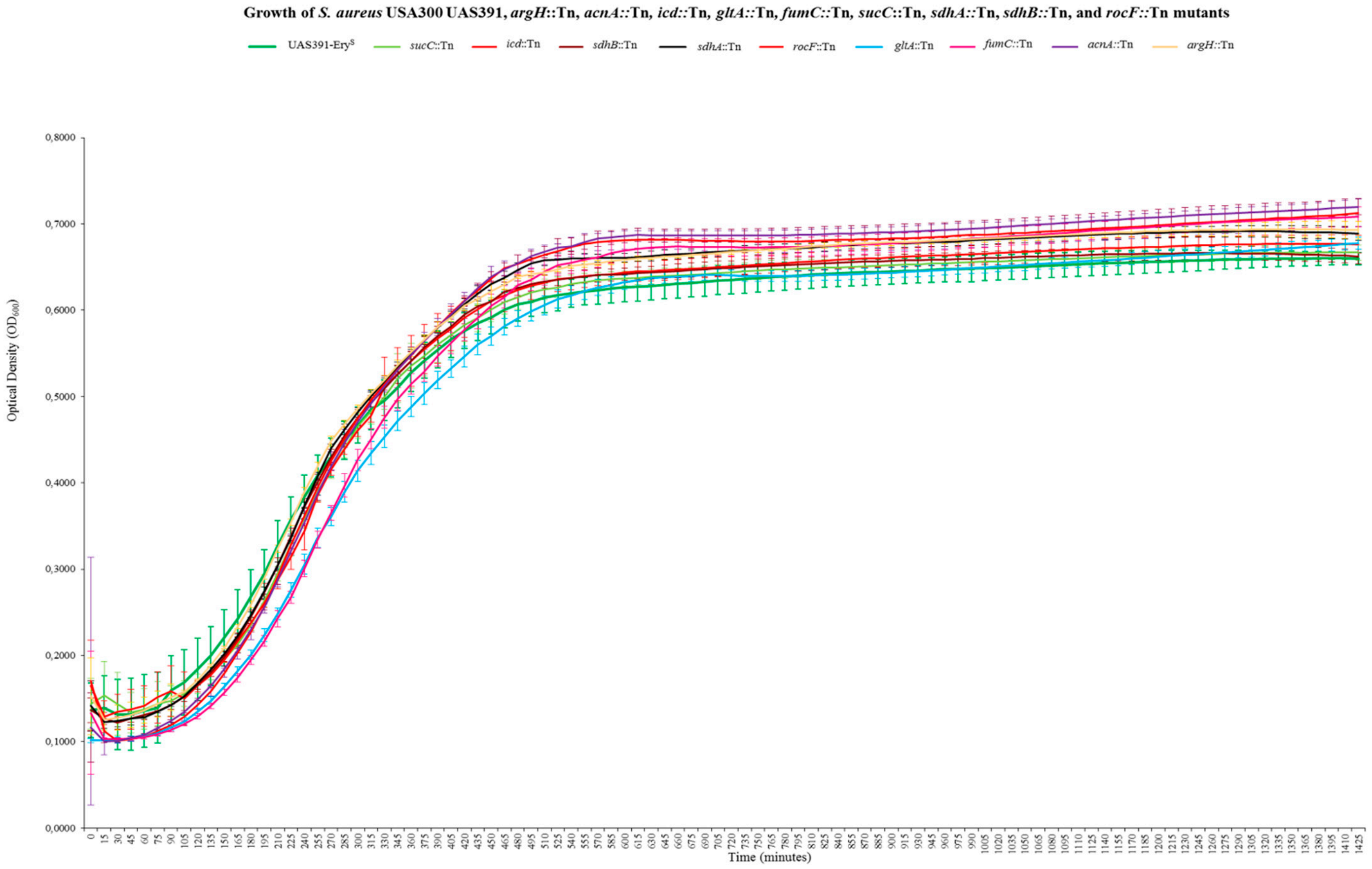

Supplementary Figure S1: Background absorption-corrected 24 h growth curves for UAS391-Ery^S as well as *argH*::Tn, *acnA*::Tn, *icd*::Tn, *gltA*::Tn, *fumC*::Tn, *sucC*::Tn, *sdhA*::Tn, *sdhB*::Tn and *rocF*::Tn mutants. Error bars in corresponding color represent the 95% confidence interval per strain.

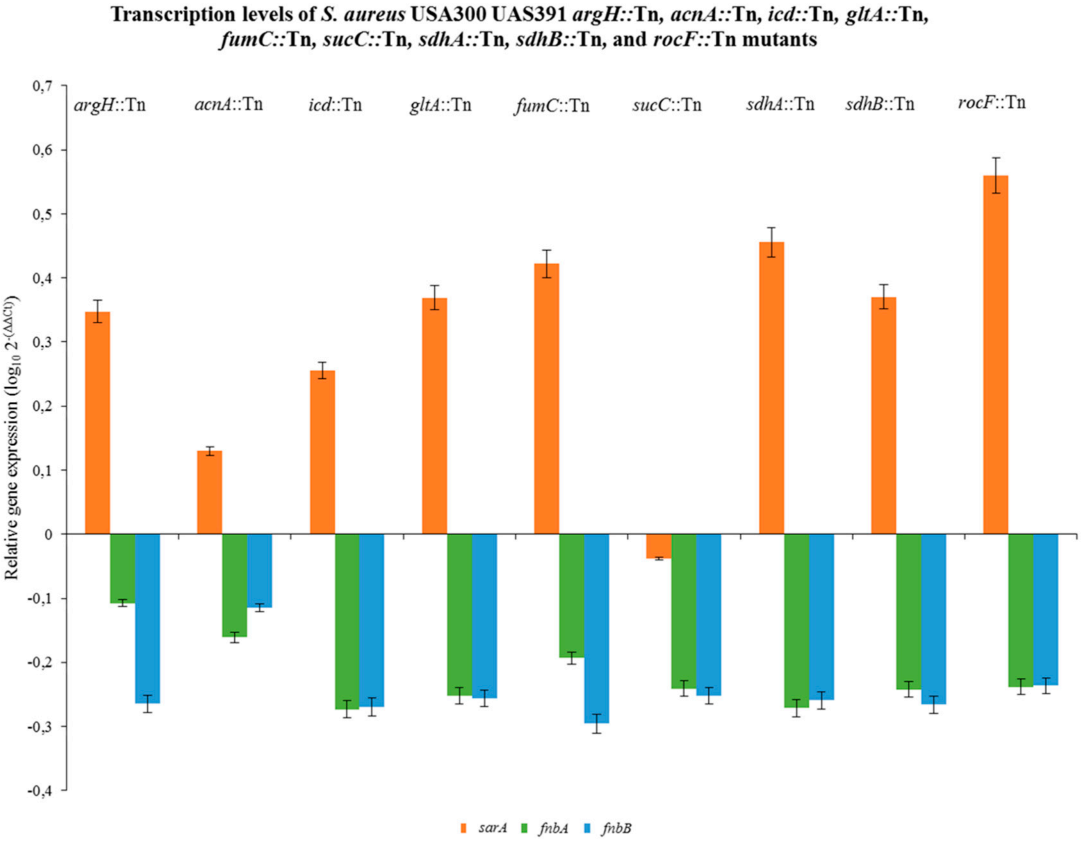

Supplementary Figure S2: Relative quantification of *fnbA*, *fnbB* and *sarA* gene expression in UAS391-Ery^S and the corresponding TCA- and urea cycle knockout mutants, normalized to *gyrB* expression and calculated using the Comparative C_t Method ($2^{-\Delta\Delta Ct}$). Gene expression in UAS391-Ery^S was taken as baseline 0.

Supplementary Table S1: Primers used during this study. Primers were designed against *S. aureus* strain USA300-UAS391 (#CP007690.1).

Gene name	ID	Sequence (5′→3′)	Product size (bp)	Reference
Primers for RT-PCR				
gyrB	GyrB-F	GTAACACGTCGTAAATCAGCG	170	[32]
	GyrB-R	CGTAATGGTAAAATCGCCTGC		
fnbB	RTFnbB-F	AGGTGCAGAAGGTCATGCAG	222	This study
	RTFnbB-R	TGATCGCTAACAGCACCAGT		
fnbA	RTFnbA-F	CCAGTACCACCTGCCAAAGA	195	
	RTFnbA-R	TCCGCCGAACAACATACCTT		
sarA	RTSarA-F	GCTGTATTGACATACATCAGCGA	250	
	RTSarA-R	CGTTGTTTGCTTCAGTGATTCG		
Primers for complementation				
pALC2073 plasmid	TetR-2	CAATGTAGGCTGCTCTACACCTAG	547	[17]
	pALC-2	GATCGGTGCGGGCCTCTTCGCTAT		

argH	ArgH-1	AGCTTGATGGTACCGAGCTCGAATTTGGAGGCTATAGCAATGAGCAATA	1807	This study
	ArgH-2	GTTGTAAAACGACGGCCAGTGAATTCTTATTGTGATAGTAATTGTTTAGCAAC		
acnA	AcnA-1	AGCTTGATGGTACCGAGCTCGAATTATGTATCAAGGGGGATCATTAAATGGCTGCAAATTTTAAAGAGCAATC	3141	
	AcnA-2	GTTGTAAAACGACGGCCAGTGAATTCTTATTGCGCTAATTTATTTCTTAAAACCATTTG		
gtlA	GltA-1	AGCTTGATGGTACCGAGCTCGAATTTAAAGGGGAAATTTATCATGGCAGAATTACAAAGAG	GltA-1 + GltA-2: 1522 GltA-1 + Icd-2: 2869	
	GltA-2	GTTGTAAAACGACGGCCAGTGAATTCTTATTTTCTTTCTTCAAGCGGGATATA		
Icd	Icd-1	AGCTTGATGGTACCGAGCTCGAATTTTGGAGGTAAAATAACTATGACTGCAGAAAAAATTAC	Icd-1 + Icd-2: 1699 GltA-1 + Icd-2: 2869	
	Icd-2	GTTGTAAAACGACGGCCAGTGAATTCTTATTTTAAATTTTTAATCAATTCATC		
rocF	RocF-1	AGCTTGATGGTACCGAGCTCGAATTTAGAGCAAAGGGGGACGCTTATGACAAAGACAAAAG	1342	
	RocF-2	GTTGTAAAACGACGGCCAGTGAATTCTTATAATAAAGTTTCACCAAAAAATGTTCCAAC		
fumC	FumC-1	AGCTTGATGGTACCGAGCTCGAATTAACAGTGATAAGGGGAGAAATTGAATGTCAGTAAGAATTGAAC	1823	
	FumC-2	GTTGTAAAACGACGGCCAGTGAATTCTTAATGAGGATCTACCATATCTTCTG		
sdhA	SdhA-1	AGCTTGATGGTACCGAGCTCGAATT AGGGGAGTGAAATTTTTATGGCAGAGAAACATC	SdhA-1 + SdhA-2: 2197 SdhA-1 + SdhB-2: 3012	
	SdhA-2	GTTGTAAAACGACGGCCAGTGAATTCTTATTTTTTACCCCCTTTAGACTTAC		
sdhB	SdhB-1	AGCTTGATGGTACCGAGCTCGAATTCTAAAGGGGGTAAAAAATAATGACTGAACAATCAGTG	SdhB-1 + SdhB-2: 1254 SdhA-1 + SdhB-2: 3012	
	SdhB-2	GTTGTAAAACGACGGCCAGTGAATTCTTATTCTACTTCATGGTCTGAACCAAAG		
sucC	SucC-1	AGCTTGATGGTACCGAGCTCGAATTAACCTAAGTAACAGGAGGATGGAAGATGAATATCCACGAGTATC	SucC-1 + SucC-2: 1605 SucC-1 + SucC-3: 2595	
	SucC-2	GTTGTAAAACGACGGCCAGTGAATTCTTATGCTTCTTTGACTAGTTTAAC		
	SucC-3	GTTGTAAAACGACGGCCAGTGAATTCTTATTTATTAACAGTTAATAATGATTC		

32. Sihto, H.M.; Tasara, T.; Stephan, R.; Johler, S. Validation of reference genes for normalization of qPCR mRNA expression levels in *Staphylococcus aureus* exposed to osmotic and lactic acid stress conditions encountered during food production and preservation. *FEMS Microbiol. Lett.* **2014**, *356*, 134–140, doi:10.1111/1574-6968.12491.
